# Measuring the Impact of Food Immunotherapy on Health-Related Quality of Life in Clinical Trials

**DOI:** 10.3389/falgy.2022.941020

**Published:** 2022-07-12

**Authors:** Melanie Lloyd, Audrey Dunn Galvin, Mimi L. K. Tang

**Affiliations:** ^1^Allergy Immunology Group, Murdoch Children's Research Institute, Parkville, VIC, Australia; ^2^School of Public Health and Preventative Medicine, Monash University, Melbourne, VIC, Australia; ^3^School of Applied Psychology, Cork University Hospital, University College Cork, Cork, Ireland; ^4^Department of Paediatrics and Paediatric Infectious Diseases, Institute of Child's Health, Sechenov First Moscow State Medical University, Moscow, Russia; ^5^Department of Allergy and Immunology, Royal Children's Hospital, Parkville, VIC, Australia; ^6^Department of Paediatrics, University of Melbourne, Parkville, VIC, Australia

**Keywords:** food allergy, immunotherapy, outcome measurement, health-related quality of life, clinical trial

## Abstract

Food allergy is a common, and often lifelong, disorder with considerable negative impact on the quality of life of those affected and their families. While several promising immunotherapies for food allergy have either been approved or are in late-phase clinical trials based on demonstrated effectiveness at inducing desensitization, evidence of benefit in terms of improving patient-centered outcomes is inconsistent. Historically, health-related quality of life has not been prioritized as an endpoint in food immunotherapy trials and, even when included, findings have been undermined by methodological limitations of the measurement instruments used and issues with data interpretation. This review highlights the importance of measuring health-related quality of life as an endpoint in food immunotherapy trials and discusses the strengths and limitations of available evidence in this regard, with a focus on the appropriate use of assessment instruments and interpretation of findings. There remains much to learn regarding the impact of food immunotherapies on patient wellbeing, both during treatment and over the longer term. Our aim is to assist clinicians, researchers, policy makers and consumers in their interpretation of the existing literature, and to promote greater scientific rigor in the design and selection of outcome measurement frameworks for future studies evaluating the efficacy of immunotherapy treatments for food allergy.

## Introduction

Food allergy is a chronic disorder that affects 10% of infants and 5–8% of children (1). Recent data from prevalence surveys and healthcare utilization also indicate a worrying increase in adult-onset food allergy, with 10.8% of participants in a US study reporting a history consistent with IgE-mediated reactions and/or a diagnosis history of food allergy–related health care (2, 3). Diagnosis is usually made in infancy and while some food allergies resolve during childhood, allergies to nuts, fish and shellfish mostly persist throughout life (4, 5). There is no cure, so management relies on strict allergen avoidance and the use of rescue medication for reactions following accidental ingestion (5). There is now strong evidence that food allergy causes psychological distress and has a severe negative impact on quality of life for the affected individual and their family, primarily driven by the fear of accidental reactions and lifestyle restrictions caused by having to avoid allergens (6, 7). Qualitative and quantitative research also shows that living and coping with food allergy may have an adverse impact on the developmental process itself (8).

To date, studies of food immunotherapy have largely focused on efficacy and safety outcomes such as desensitization (increase in reaction threshold), remission, and treatment-related adverse events (9–11). While crucial to assessment of intervention efficacy, these fail to capture the effects of treatment on patient-important factors impacted by food allergy such as psychological and emotional wellbeing, social interaction and participation, which are central to life-long management of chronic illness. Without holistic assessment of outcomes, particularly from the perspective of the patient and over the longer term, it is difficult to determine both the true value of an intervention and patients who are most likely to benefit. Moreover, cost-effectiveness analyses will be driven primarily by quality of life impact and reactions (12). If the goal is to improve the lives of individuals affected by food allergy and their families, it is crucial to address the psychological and quality of life impacts of food allergy. It is incumbent on health care professionals, government, and patient advocacy groups to prioritize health-related quality of life (HRQL) when evaluating novel therapies.

The importance of patient-centered outcomes for driving value-based decision making in both health research and routine clinical practice is now well-established (13, 14). To be patient-centered, an outcome must be important and meaningful to patients and caregivers (15), and self-reported questionnaires are widely used to capture the patient “voice” in a standardized way. Validated patient-reported instruments are powerful in their ability to quantify change, facilitate sample size calculations and define minimal clinically important differences (16). Subjective self-reported instruments can also be supplemented by objective clinical measures that capture outcomes meaningful to patients (17).

Food allergy-specific HRQL questionnaires have been developed and validated for different age groups (child, adolescent, and adult) and perspectives (allergy sufferer, caregiver) (7, 18, 19). Through application of these instruments, our understanding of the lived experience of food allergy has greatly improved over the past decade (20). However, adoption of patient-centered outcomes in food immunotherapy interventional trials has been inconsistent (9, 21, 22). Considerable heterogeneity in the use of endpoints for food allergy trials has made compiling and comparing the efficacy of different treatments difficult (10, 22, 23). The double-blind placebo-controlled food challenge is the gold standard for determining ability to safely consume a food allergen (10), however, these have no validated utility for predicting the frequency and severity of allergic reactions in the real world (21). At the same time, there are significant gaps in the measurement and reporting of data in relation to reactions and allergen ingestion following treatment for food allergy. Evidence that novel immunotherapies improve HRQL is limited (11, 21) and in particular, there are few long-term studies assessing for sustained improvements (22, 23).

As the primary burden on patients living with food allergy is reduced quality of life, treatment success in trials should be defined not only by clinical outcome (desensitization, remission) and safety but also by improved HRQL. It is likely that interactions exist between clinical outcome, safety *and* HRQL, which may provide a new understanding of “benefit” for the patient and the caregiver. Sim et al. proposed a core outcomes set for food allergy clinical trials that puts patient-centered outcomes front and center, highlighting how failure to harmonize outcomes measurement using validated instruments has limited our understanding of whether food allergy treatments improve the lives of allergy sufferers (9). This review discusses the methodological limitations and considerations impacting the interpretation of HRQL outcomes in clinical trials of food immunotherapies, to assist clinicians, researchers, policy-makers, and consumers when critiquing and comparing available evidence on treatment effectiveness.

## Methodological Limitations Impacting the Interpretation of HRQL Outcomes in Food Immunotherapy Trials

### Absence of Blinding, Placebo Control or Randomization

Involvement in a clinical trial *per se* is known to be associated with general improvements in patient outcomes (24). Inclusion of a placebo control with blinding to treatment arm is therefore essential to account for universal improvements associated with participation in an intensive monitored food immunotherapy regimen. Several studies have reported improvement in HRQL for placebo patients participating in food immunotherapy trials (25–27), presumably due to the increased clinical support received during the trial. Blinding to treatment arm is not possible in studies that utilize an observation control arm. To date, only two blinded randomized controlled trials have compared HRQL between food immunotherapy (active intervention) and placebo arms, *and* included long term follow up of HRQL in both the active and placebo arms (25, 28, 29). Several open label, cohort-controlled, and randomized controlled studies have reported improvement in HRQL compared to baseline in patients who received oral immunotherapy (OIT), however, the lack of a blinded comparison group in these studies limits the strength of findings since it is impossible to exclude a placebo effect (30–36). A study of peanut epicutaneous immunotherapy (EPIT) reported HRQL improvement in children who achieved an increase in reaction eliciting dose following 24 months of EPIT compared with a control group who received placebo for 12 months followed by EPIT for 12 months (27). However, no data was presented comparing the EPIT-treated vs. placebo-treated groups. Future studies evaluating novel immunotherapies should include HRQL as an *a priori* endpoint in blinded randomized trials.

### Lack of Comparability Between Instruments

Multiple different generic and disease-specific HRQL instruments have been applied in food allergy interventional trials (22). Generic instruments enable the comparison of HRQL outcomes between diseases and populations, while disease-specific questionnaires can provide a nuanced illustration of the day-to-day experience of living with a particular medical condition, and may be more sensitive to small changes in HRQL that occur in response to intervention (37). This is particularly relevant in food allergy because generic instruments that focus on pain or functional deficits lack the specificity to capture the psychological burden of allergen avoidance (the mainstay of food allergy management) when symptoms are rare (7). Identification of food-allergy specific symptoms and psychosocial factors may also be important when differentiating between the impact of different allergens (e.g., milk vs. peanut) or mediation pathways (IgE or non-IgE mediated).

While each has specific strengths and weaknesses, heterogeneity in the selection of instruments prevents direct comparison of outcomes between studies (9). Commonly used instruments measure different constructs such as symptoms, emotional wellbeing or patient satisfaction (9, 38). Even where instruments measure similar constructs, differences in scoring methods, magnitude of the minimal clinically important difference, or validation cohort characteristics may preclude direct comparisons. Furthermore, while evidence of reliability and validity exists for most, none of the existing disease-specific instruments that utilize parent/caregiver-report completely satisfy established instrument development guidelines (38). A standardized measurement framework that incorporates patient-centered outcomes, together with agreed definitions of constructs, scales and timeframes, would allow for the comparison of efficacy of food allergy treatments between centers, trials, and/or settings. The core outcomes set proposed by Sims et al. provides a starting point for investigators, and an upcoming systematic review should further address issues surrounding the appropriateness of alternative disease-specific instruments (9, 39).

### Longitudinal Measurement of HRQL

Application of age group-specific instruments in longitudinal studies of pediatric populations also presents methodological challenges. Bio-psychosocial development during childhood means that dimensions relevant to HRQL change rapidly with age. The developmental process and attainment of important life milestones necessitate variation in the way questions are framed and may impact the outcome of interest independently of the treatment interventions received (18, 37). While short-term longitudinal validity of the widely used age-specific Food Allergy Quality of Life Questionnaires (FAQLQ) has been established (40), further research is required to determine whether transition from the FAQLQ-Child to FAQLQ-Teenager to FAQLQ-Adult forms, when administered to a single participant, can support valid comparison of HRQL over time.

### Discordant Child and Caregiver HRQL Scores

The availability of both self-rated and caregiver-proxy-rated instruments has highlighted the relevance of perspective when assessing HRQL in food allergy. Multiple studies have reported divergent HRQL scores by parent-proxy compared to child-report (27, 33, 41–43), with various explanations for discrepancies suggested. For example, study participation may be a more positive experience for caregivers, with an associated reduction in risk and uncertainty enhancing parent-reported HRQL (33, 41). Children undergoing treatment, however, are required to consume foods they may dislike and are preoccupied more with immediate symptoms and experiences, rather than long-term benefits (41). Differences in parent vs. adolescent perception of allergy severity and illness comprehension have also been proposed to explain discrepancies (44). Parents may more reliably remember certain events (and their impact) than children and/or they give more weight to certain outcomes in their assessment of the child's HRQL than their children do themselves (42). In light of the lack of consensus or guidelines around when and at what age self-report and proxy-report administrations should be used, where feasible, both self- and caregiver proxy-reported HRQL should be collected and presented, to provide a more holistic view of impact and outcome (33).

## Considerations When Interpreting HRQL Measurements

### Interaction Between Allergic Status and HRQL

The ability to freely consume an allergen as desired, and the quantity/frequency of ingestion are likely to be strong drivers of improved HRQL in food allergy. Free ingestion depends on the clinical outcome of treatment, as this determines the need for continuing treatment and allergen avoidance (45). Desensitization refers to an increase in reaction threshold that is only maintained though continuous treatment (or allergen exposure) (46). Therefore, a patient who is desensitized with immunotherapy gains protection against allergic reactions to accidental allergen intake, but must continue with both daily treatment indefinitely and strict allergen avoidance to maintain this protection. Remission, on the other hand, refers to an absence of clinical reactivity (e.g., passing a diagnostic food challenge) after treatment has been discontinued for a period of time (e.g., weeks or months). Remission allows patients to stop treatment and eat the allergen as part of their usual diet, removing the need for avoidance (47).

There is limited data evaluating the impact of different clinical health states (allergic, desensitized without remission, and remission) on HRQL and other important patient-centered outcomes. Several studies suggest an interaction between allergy status and HRQL (25, 27, 28). The PPOIT-003 study included a 12-month follow-up period which provided a vital snapshot of the real-world post-treatment scenarios for children who achieved remission, desensitization alone (without remission) or remained allergic, allowing direct assessment of these interactions (28). Children with remission were eating peanut as desired, while desensitized children were taking a daily treatment dose of peanut while avoiding all other peanut, and allergic children were avoiding all peanut. The vast majority of children with remission were having regular exposure to substantial amounts of peanut, with 94% eating peanut at least monthly, and 80% eating 600 mg peanut protein or more at a single ingestion (28). Children in remission had significantly greater improvement in HRQL compared with children who were only desensitized (without remission), suggesting that remission is a better outcome for patients (28). Children in remission were also shown to have significantly greater improvement in HRQL compared to children who remained allergic and continued with allergen avoidance (28). Based on these findings, the lack of conclusive evidence that OIT improves HRQL in other studies may relate to patients only achieving desensitization, rather than remission (26, 33). Other than the PPOIT-001 and PPOIT-003 studies, only three placebo-controlled randomized clinical trials of peanut oral immunotherapy completed to date (to our knowledge) have measured the remission endpoint, however none of these reported HRQL outcomes (48–50).

The level of desensitization that is achieved following immunotherapy may also impact on the degree of HRQL benefit as there may be perceived and real differences in the degree of protection achieved. For example, low dose peanut OIT (300 mg peanut protein) and peanut EPIT have been shown to desensitize patients to 1,000 mg peanut protein (26), which should protect against limited amounts of peanut cross contamination but would not prevent reactions to larger amounts of peanut ingestion (e.g., accidental ingestion of a food where peanut is an ingredient). High dose OIT treatments have been developed to provide higher level protection (28, 51), and were shown to provide significant and lasting HRQL improvement (28). Interestingly, HRQL improvement increased over time, presumably as the lifestyle benefits of clinical remission are realized in the real world, and HRQL improvement was specifically linked to the amount (both quantity and frequency) of peanut ingested (25, 29). Taken together, these findings suggest that HRQL improvements are driven by the lifestyle benefits of free consumption without the need for continuing daily treatment, and emphasize the importance of considering clinical outcome when evaluating HRQL impacts of novel therapies.

### Timing of Outcome Measurement

The timing of assessment relative to clinical outcome achieved is likely to influence HRQL impact of an intervention. As alternative delivery methods for immunotherapy are developed (e.g., oral, epicutaneous, sublingual), it will be important to distinguish between these in terms of HRQL impact during the treatment phase. Burden of treatment may differ between modalities with regard to protocol rigidity, clinic visit schedule, treatment-related symptoms and adverse events, and the total duration of time for which treatment must continue, all of which may impact HRQL for patients even if clinical outcomes are similar between treatments. Measurement of patient-centered outcomes both during and after treatment allows specific examination of the impact of immunotherapy treatment *per se*, aside from the subsequent post-treatment experience (25). Emerging evidence suggests that the up-dosing phase of OIT is associated with reduced HRQL, possibly due to frequent reactions and symptoms (25, 28, 32).

Long-term follow-up post-treatment is important to determine if HRQL benefits are maintained, lost or increased, as participants adjust to their altered allergy status (29). Neuro-psychological research shows that threat perception changes over time as memory of the threat is either reconsolidated (e.g., through recurrent reactions) or extinguished (e.g., through regular safe consumption of the culprit allergen) (52). It is conceivable that over time, remission may offer further improvement in HRQL, as reported previously, whereas desensitization alone (which is associated with continuing and frequent treatment-related reactions) does not (29). Measurement of HRQL at multiple intervals during the trial and beyond is therefore important for systematic analysis and modeling of antecedent factors, mediators, and outcomes to fully understand the benefits of treatment.

### The Impact of Patient Factors on HRQL Outcomes

It is plausible that different sub-groups will respond differently to food allergy treatments in terms of HRQL outcomes. Patient characteristics, for example age of child, severity of prior reactions, and type or number of food allergies have been shown to be associated with HRQL (32, 37, 53–55), though the interaction of these with treatment effect in immunotherapy remains unclear (37, 56). Parental self-efficacy in managing food allergy is also an important contributor to caregiver-reported HRQL (55).

To date, there has been little exploration of psychological factors correlated with HRQL outcomes (e.g., anxiety, health beliefs, risk perception, information processing, coping behaviors), and the impact of these on treatment success (18, 57). The causal pathways for these factors on HRQL status also remain to be determined. Environmental factors, including community and socioeconomic variables that can enhance or diminish the effect of a treatment on targeted therapeutic outcomes, should also be explored in relevant pathway models.

Improved understanding of the interaction between patient characteristics and predicted HRQL outcomes from therapy will assist clinicians to discuss potential benefits and harms, and help families choose whether immunotherapy is right for them. It is plausible that many patients and their families may prefer to continue avoiding an allergen when informed of the risk of ongoing reactions, burden of treatment adherence and relative likelihood of long-term improvements in HRQL. Identification and standardization of predictors could optimize treatment by enabling tailored treatment approaches that are matched to a particular patient profile (6, 37).

## Discussion

Patient-centered outcomes such as HRQL have been identified and promoted as a priority endpoint when evaluating effectiveness of a food immunotherapy, however, there remain many methodological limitations to their use and interpretation. Given the limitations of food challenges in predicting reactions outside of a controlled clinical setting (21), additional objective metrics of health benefit and harm have been proposed by key academic institutes and regulatory bodies (18, 46, 58), including patterns of intentional ingestion of the culprit food, as well as number, frequency and severity of allergic reactions, which have all been shown to correlate with HRQL (9). Key priorities for the field include reaching consensus on a core outcomes set for both research and routine clinical practice, as well as establishment of an accepted timeframe for outcomes measurement before, during and after treatment. HRQL in particular is important for evaluating treatment impact, acceptability, cost-effectiveness, and informing patient-centered clinical decision making (27). The limitations of existing HRQL measurement frameworks must be addressed if priority knowledge gaps, such as identification of patient factors impacting treatment response and interaction of the child development process and psychological phenotypes with HRQL outcomes, are to be explored.

Having standardized definitions and measurement approaches for adverse events, severe reactions and clinical outcomes would also significantly aid comparability and allow for more targeted patient support and regulatory guidance (6). Given the early evidence that achieving remission offers significant HRQL benefits for patients that are greater than with desensitization alone, more work is required to reach consensus on the appropriate clinical definition of the remission endpoint. In particular, the length of time a patient should be off treatment before assessing for remission is unclear. While adequate time should be allowed to exhaust residual desensitization effects, extended avoidance of allergen exposure may lead to regression of newly established (unstable) immune changes (28). As some amount of continued allergen intake is considered necessary to support consolidation of a newly rewired immune network underpinning the remission state, an earlier remission test would result in a greater proportion of patients identified as having attained remission and able to commence free peanut intake to support sustained redirection of immune responses. Furthermore, there is no clear definition of the dose of allergen that must be tolerated to confirm remission. We propose that the challenge amount should be equivalent to a standard diagnostic challenge since passing a diagnostic challenge after treatment cessation justifies a label of clinical remission.

By considering the timing and selection of patient-centered outcome measures, trial investigators would be able to generate data that can inform and guide clinical practice. It is vital that outcomes are captured in the real world beyond the tightly controlled clinical trial environment, both through long-term follow-up and Phase 4 trials. A peanut oral immunotherapy is now approved for use in clinical practice in the US, Europe and UK (59, 60), and unregistered OIT using commercially available food sources is offered in many countries, despite limited understanding of how these treatments benefit patients (61). With increasing application of food immunotherapy, there will be opportunity for evaluation of treatment benefits in real world settings (45), and patient-centered outcomes should be integral to this evaluation.

Our understanding of HRQL in food allergy is improving but there is much more to learn regarding the impact of different interventions on patient wellbeing during treatment and over the longer term. High quality interventional trials that consistently measure these outcomes in a standardized way are vital to fully evaluate the real-world benefit to patients of novel treatments.

## Author Contributions

This article was conceived jointly by ML and MT. ML led the development of the preliminary draft. All authors reviewed the content for accuracy and completeness. All authors contributed to the article and approved the submitted version.

## Funding

The Murdoch Children's Research Institute was supported by the Victorian Government's Operational Infrastructure Support Programme.

## Conflict of Interest

AD reports having received consultant fees from Aimmune Therapeutics, DBV Technologies, and Nestle (research grant and advisory panel). MT declares consultant fees from Pfizer and Abbott Nutrition; inventorship on patents covering PPOIT; employee and scientific founder of, and holds share interest and options in, Prota Therapeutics; membership of the Medical Advisory Board of Anaphylaxis & Anaphylaxis Australia and past membership of the Board of Directors of the World Allergy Organization (WAO, ended 2019); membership of expert committees of the American Academy of Allergy Asthma and Immunology, Asia Pacific Association of Allergy Asthma and Clinical Immunology, Australasian Society of Clinical Immunology and Allergy, WHO, and past membership of the International Union of Immunological Societies (ended 2019). The remaining author declares that the research was conducted in the absence of any commercial or financial relationships that could be construed as a potential conflict of interest.

## Publisher's Note

All claims expressed in this article are solely those of the authors and do not necessarily represent those of their affiliated organizations, or those of the publisher, the editors and the reviewers. Any product that may be evaluated in this article, or claim that may be made by its manufacturer, is not guaranteed or endorsed by the publisher.
